# Overlapped Partitioning for Ensemble Classifiers of P300-Based Brain-Computer Interfaces

**DOI:** 10.1371/journal.pone.0093045

**Published:** 2014-04-02

**Authors:** Akinari Onishi, Kiyohisa Natsume

**Affiliations:** Graduate School of Life Science and Systems Engineering, Kyushu Institute of Technology, Kitakyushu, Fukuoka, Japan; University of Catania, Italy

## Abstract

A P300-based brain-computer interface (BCI) enables a wide range of people to control devices that improve their quality of life. Ensemble classifiers with naive partitioning were recently applied to the P300-based BCI and these classification performances were assessed. However, they were usually trained on a large amount of training data (e.g., 15300). In this study, we evaluated ensemble linear discriminant analysis (LDA) classifiers with a newly proposed overlapped partitioning method using 900 training data. In addition, the classification performances of the ensemble classifier with naive partitioning and a single LDA classifier were compared. One of three conditions for dimension reduction was applied: the stepwise method, principal component analysis (PCA), or none. The results show that an ensemble stepwise LDA (SWLDA) classifier with overlapped partitioning achieved a better performance than the commonly used single SWLDA classifier and an ensemble SWLDA classifier with naive partitioning. This result implies that the performance of the SWLDA is improved by overlapped partitioning and the ensemble classifier with overlapped partitioning requires less training data than that with naive partitioning. This study contributes towards reducing the required amount of training data and achieving better classification performance.

## Introduction

The P300 is a component of an event-related potential (ERP) in a non-invasive scalp electroencephalogram (EEG) that was discovered by Sutton *et al.* [1]. The P300 appears as a positive peak approximately 300 milliseconds (ms) after a rare or surprising stimulus. The P300 is elicited by the oddball paradigm: rare (target) and non-rare (non-target) stimuli are presented to a participant, and then he/she counts the occurrence of the target stimuli silently. The P300 can be seen in the ERPs corresponding to the target stimuli. Visual and auditory stimuli have often been used to elicit the P300 [2], [3]. Currently, the P300 is used in brain-computer interfaces (BCIs) for controlling devices.

The P300 was first utilized for spelling out letters by Farwell and Donchin in 1988 [4]. They proposed a BCI system that typed letters according to the detected P300 elicited by the visual target stimuli, referred to as a P300-based BCI or a P300 speller. The P300-based BCI can control not only a speller but also a wheelchair [5], [6], computer-mouse [7], web browser [8], virtual reality system [9], game [10], or smart phone [11]. Since the BCI does not depend on muscle activity, it constitutes a new interface that will provide a better quality of life for patients disabled by neuromuscular diseases, such as amyotrophic lateral sclerosis (ALS) [12]. The interface, classification methods, and their extensions have been studied for more than 20 years (e.g., [13]–[15]).

Stepwise linear discriminant analysis (SWLDA) has been widely used as a standard classification algorithm for the P300-based BCI [16]–[19]. Farwell and Donchin first proposed the SWLDA, together with the entire classification protocol for P300 [4]. Schalk *et al.* proposed a general-purpose BCI system, named BCI2000, in which the P300-based BCI was implemented together with the SWLDA [20]. Krusienski *et al.* compared the classification algorithms for BCI [21]. Specifically, they compared the classification accuracy of Pearson's correlation method, linear discriminant analysis (LDA), SWLDA, linear support vector machine (SVM), and Gaussian kernel SVM. The results showed that LDA and SWLDA achieved a better performance than the others. Blankertz *et al.* proposed an LDA with shrinkage for P300-based BCI that yielded a better performance than SWLDA when a small amount of training data were given [22].

Ensemble classifiers are among the most powerful classifiers for the P300-based BCI; however, they were developed and evaluated using a relatively large amount of training data. The ensemble of SVMs proposed by Rakotomamonjy and Guigue won the BCI competition III data set II that contains a huge amount of training data (15300 ERP data) [23]. They applied the ensemble classifiers to reduce the influence of signal variability using the classifier output averaging technique [24]. Salvaris *et al.* compared the classification accuracies of ensemble LDA and ensemble SVM classifiers using the BCI competition III data set II and BCI competition II data set IIb (7560 training data) [25]. They also employed an ensemble of six linear SVM classifiers and evaluated classification accuracies using their own data by 16-fold cross-validation [26]. An ensemble SWLDA classifier was first proposed by Johnson *et al.* and evaluated on their own P300-based BCI data (6480 training ERP data) [27]. Arjona *et al.* evaluated a variety of ensemble LDA classifiers using 3024 training data [28].

In online (real-time) P300-based BCI experiments, a smaller amount of training data compared to the training data used in the BCI competition III data set II and BCI competition II data set IIb tended to be used. Townsend *et al.* recorded 3230 ERP training data for a row-column paradigm and 4560 ERP training data for a checkerboard paradigm [15]. Guger *et al.* evaluated the online performances of P300-based BCI, where LDA was trained on 1125 ERP training data [29]. The EEG data are usually high dimensional and the target training data that contain P300 were rare (e.g., 1/6) and have different statistical property from the non-target data. In other words, researchers must address the class imbalance problem [30] that is severely prone to overfitting. Thus the thousands of training data can be considered small in this field. To be practical, the amount of the training data should be small in order to reduce the training time [21]. However, most of the studies on the ensemble classifiers for the P300-based BCI did not evaluate the classification accuracy using a practical amount of training data, e.g., less than 1000 ERP data.

In an online experiment where less than 1000 training data are given, the ensemble classifier may not perform well because of its method of partitioning training data. Most ensemble classifiers employ naive partitioning that divides training data into partitions by sets of data associated with a target command [23]. According to the use of the naive partitioning, training data were partitioned without overlaps. Johnson *et al.* also employed the same partitioning method [27]. Due to the naive partitioning method, however, each weak learner in the ensemble classifier is trained on a smaller amount of training data than a single classifier. In addition, the dimension of the EEG data is usually high. In such cases, classifiers are prone to overfitting [32]. Thus, the classification performance of the ensemble classifiers may deteriorate when the amount of training data is small and ensemble classifiers should therefore be evaluated when less than 1000 training data are given.

To develop a better classifier that requires less than 1000 training data, we propose a new overlapped partitioning method to train an ensemble LDA classifier, which we evaluated when 900 training data were given. The overlapped partitioning allows a larger amount of training data to be contained in a partition, although a part of the training data were reused. The proposed classifiers were evaluated on our original P300-based BCI data set and the BCI competition III data set II, using small (900) training data and large (over 8000) training data. One of three conditions for dimension reduction was applied: the stepwise method, principal component analysis (PCA) or none. Our objective was to clarify how the ensemble LDA classifiers with overlapped or naive partitioning and the single LDA classifier performed when 900 training data were given.

Overlapped partitioning is a new partitioning method that is applied in the training of an ensemble classifier, and is designed such that it will be suitable for application in P300-based BCI. When we evaluated the performance of the new method, we also assessed the influences of dimension reduction methods. The algorithms were first compared under the condition that 900 training data were used, which were the smallest amount of data used to date for the evaluation of ensemble classifiers for P300-based BCI. In addition, the influence of the degree of overlap used in the ensemble classifier with overlapped partitioning was demonstrated for the first time. We consider that the overlapped partitioning is essential to implement the ensemble classifiers in an online system. This study contributes towards reducing the required amount of training data and achieving better classification performance in an online experiment.

## Methods

### Ethics Statement

This research plan was approved by the Internal Ethics Committee at Kyushu Institute of Technology. The possible risks, mental task, and approximate measurement time were explained to all participants. In addition, all participants gave their written informed consent before participating in this experiment.

### Experimental Design

**Figure 1:**
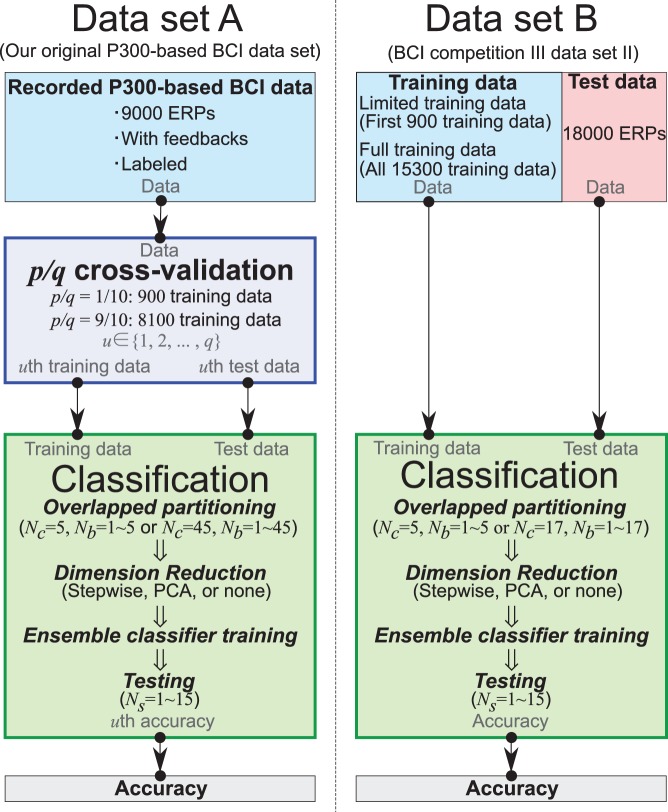
Experimental design. We analyzed two P300-based BCI data sets A and B respectively. Data set A was recorded in this online experiment. The recorded data set A is divided into 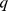 pairs of training and test data by 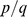 cross-validation (see Figure 4). Then the classification is performed for all pairs to compute the classification accuracy (see Figure 5). The overlapped partitioning is employed to train ensemble classifiers. Data set B (BCI competition III data set II) contains separated training data and test data. The data set was also classified by the proposed classifiers.

Ensemble classifiers with the proposed overlapped partitioning were evaluated on our original P300-based BCI data set (data set A) and BCI competition III data set II (data set B ) as shown in Figure 1. The primary objective is to clarify how the overlapped partitioning for ensemble classifiers influences the classification accuracy. The second objective is to confirm how the three conditions for dimension reduction (stepwise method, PCA, or none) improve classification performances.

### Data Set A: Our Original P300-based BCI Data Set

To evaluate ensemble classifiers, we recorded EEG data using an online P300-based BCI, and then computed the classification accuracy offline. During the EEG recording, visual stimuli were provided to the participant. At the same time, the participant performed a mental task. The recorded signals were amplified, digitized, and then preprocessed before a letter was predicted. Our data contains P300-based BCI data from 10 participants that can be used for better statistical analysis. Parameters used in the stimulus and the recording method of data set A are summarized in Table 1.

**Table 1:** Parameters of stimulators, data acquisition, and preprocessing methods for data sets A and B.

	Data set A	Data set B
#letters	36	36
#row	6	6
#column	6	6
#intensification sequence	15	15
Intensification duration (ms)	100	100
Blank duration (ms)	75	75
Target presentation duration (s)	3	2.5
Feedback presentation duration (s)	1	2.5
#participants	10	2
#recorded letters	50	training:85, test:100
#channels	8	64
Sampling rate (Hz)	128	240
Bandpass filter (Hz)	0.11–30	0.1–60
ERP buffer length (ms)	700	700
Baseline buffer length (ms)	pre-100	pre-100
Moving average (window size)	3	18
Downsampling (Hz)	43	20

#### Participants

Eleven healthy participants (ten males and one female aged 22–28 years old) participated in this study. They had no prior experience of controlling P300-based BCI. During the experiment, we checked the participants' obtained waveform as well as their health status. However, one male participant could not complete the task due to sickness. Thus, we finally analyzed data from ten participants in offline analysis.

#### Devices

**Figure 2:**
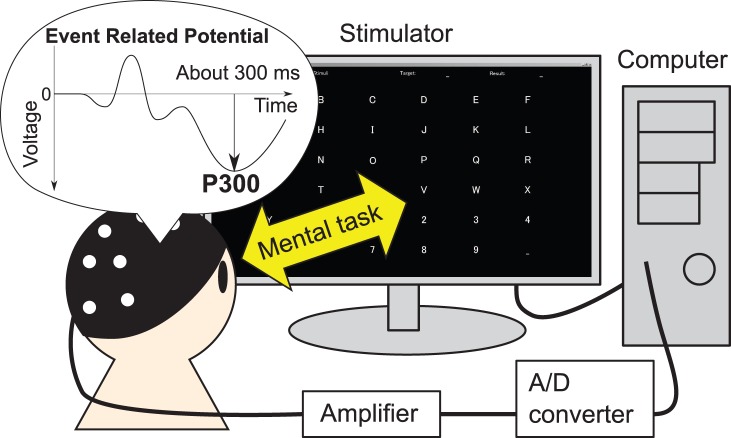
Structure of the P300-based BCI system. A target letter is presented to a participant, then letters on the stimulator are intensified by row or by column. The participant must do a mental task: silently count when the target letter is intensified. During this, the event-related potentials (ERPs) that contain the P300 component are recorded from the scalp. The signals are amplified, digitized, then stored in a computer. After finishing all intensifications, the signals were processed to predict a letter, then the feedback is displayed.

The P300-based BCI consisted of a stimulator, amplifier, A/D converter, and computer as shown in Figure 2. EEG signals were recorded at Fz, Cz, P3, Pz, P4, PO7, Oz, and PO8 scalp sites according to the international 10–20 system, which is the alignment commonly used for P300-based BCI [9]. The ground electrode was located at the AFz site and the reference electrodes were located on the mastoids. The EEG signals were filtered (0.11–30 Hz band-pass filter) and amplified 25000 times with a BA1008 (TEAC Co. Ltd., Japan). Then, the signals were digitized by an AIO-163202FX-USB analog I/O unit (CONTEC Co. Ltd., Japan). The sampling rate was 128 Hz. The P300-based BCI was implemented by MATLAB/Simulink (Mathworks Inc., USA). The recorded signals were analyzed offline using MATLAB. Stimuli for the P300-based BCI were presented on a TFT LCD display (HTBTF-24W, 24.6 inches wide with 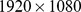 dpi; Princeton Technology Ltd., Japan) located 60 cm in front of the participant.

#### Stimuli

**Figure 3:**
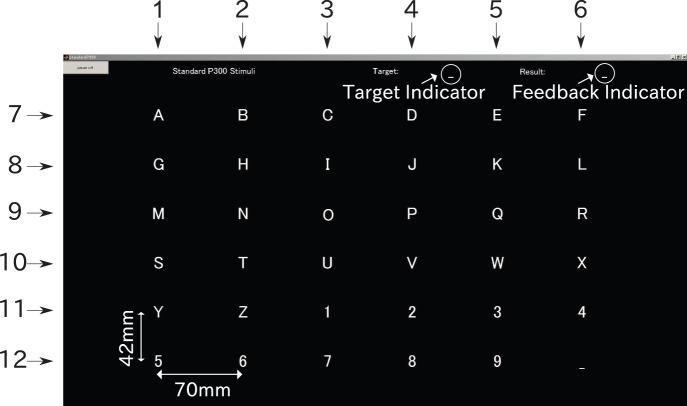
Stimulator for the P300-based BCI. It has 36 gray letters that form a matrix in the center. Each column of the matrix is numbered 1–6 and each row 7–12. A target letter is provided at the top center of the stimulator and the predicted letter is shown at the top right as feedback in test sessions.

We employed most of the parameters of the stimulator that were used in the BCI competition III data set II [23]. The stimulator of the P300-based BCI consists of 36 gray letters that form a 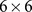 matrix, a target indicator, and a feedback indicator (see Figure 3). All the columns and rows of the matrix were numbered to manage intensifications and for the subsequent prediction of a letter. The set of column numbers was 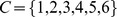, while the set of row numbers was 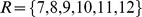. In addition, a set of all the intensifications was 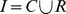. A row or a column of gray letters in the matrix turned white for 100 ms (intensification duration), and then changed to gray again for 75 ms (blank duration). At least 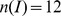 intensifications were required to identify an input letter out of the 36 letters. This is called a sequence. One row or column in a sequence was selected by a random permutation. The number of intensification sequences 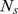 was fixed to 15 in the online experiment (i.e., 180 intensifications), while 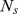 was varied from 1 to 15 in the offline analysis.

#### Preprocessing

EEG data were preprocessed for both online recording and offline analysis. The data were trimmed from the beginning of each intensification to 700 ms (8 channels×89 samples). Each 100 ms pre-stimulus baseline was subtracted from the corresponding ERP data. Subsequently, ERP data were smoothed (using a moving average with a window size of 3 ), downsampled to 43 Hz (8 channels×30 samples), and vectorized (240 channels×samples).

#### Sessions and a mental task

EEG data of P300-based BCI were recorded through a training session and ten test sessions, where only the data in the test sessions were evaluated by our proposed 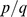 cross-validation in the offline analysis. In each session, a participant was required to spell out five letters using the P300-based BCI. A target letter to be inputted was selected randomly by the system. Thus the 900 ERPs (5 letters × 1 session × 12 intensifications × 15 sequences) were recorded in the training session and 9000 ERPs (5 letters × 10 sessions × 12 intensifications × 15 sequences) for test sessions. A target letter was displayed for 3 s, and then intensifications were presented. The participant was asked to perform the oddball task to elicit P300: the participant had to focus on the cued target letter and count silently when the letter was intensified. During the sessions, observed EEG data were recorded. In the training session, the feedback was not displayed. In the test sessions the feedback was shown in the feedback indicator for 1 s at the end of all 15 intensification sequences for the target letter. The online feedback was computed using the single LDA classifiers [21] and was presented to the participants in order to confirm whether the participant conducted the mental task appropriately in the test sessions. The feedback of success or failure also contributes to motivate participants [33], even though presenting feedbacks does not improve the classification accuracy of P300-based BCI [34]. In addition, the feedback is essential for participants to acquire the appropriate mental task [35]. Also an experimenter confirmed the feedback to make sure that the appropriate classification performance were observed using LDA. All the previous data gathered before the current session were used for learning the classifier in the online recording.

### Data Set B: BCI Competition III Data Set II

We also evaluated the proposed ensemble classifiers using BCI competition III data set II because many novel and traditional BCI algorithms have been evaluated using this data set. Since the competition data set contains a large amount of training data, we evaluated the classification performance using limited training data (900 ERPs) in addition to the full training data (15300 ERPs). Parameters used in the stimulus and data recording of the data set B are also summarized in Table 1.

#### Overview of the data set and stimulator

The data set contains EEG data for participants A and B. The EEG data were recorded from 64 channels. The recorded signals were bandpass filtered (0.1–60 Hz) and digitized at 240 Hz. The same procedure of intensifications and mental tasks for data set A was also applied to the data set B. The differences in the stimulators between data sets A and B were in the size, the font and the brightness of letters, horizontal/vertical distances of letters, and the method of presenting the target and feedback letters. It should be noted that the target and feedback presentation times were different between these two data sets, though these parameters were not directly related to the offline analysis. The data set contains EEG data corresponding to 85 target letters for training (85 letters × 12 intensifications × 15 sequences = 15300 ERPs) and EEG data of 100 target letters for testing (18000 ERPs) for each participant. A more detailed description of the data set can be found in [36].

#### Preprocessing

The same preprocessing method was used for data sets A and B; however different parameters were employed because the sampling rate and the number of channels for data set B were larger than those for data set A. All 64 channels data were used for the offline analysis. The data were trimmed from the beginning of each intensification to 700 ms (64 channels × 168 samples). Each 100 ms pre-stimulus baseline was subtracted from the ERP data. ERP data were smoothed (using moving average with window size = 18), downsampled to 20 Hz (64 channels × 14 samples), and vectorized (896 channels×samples). The vectorized data are handled as feature vectors in the classification.

### Ensemble classifiers with overlapped partitioning

The ensemble classifier divides given training data into partitions, then those partitions were used to train multiple classifiers in the ensemble classifier. The classifier in the ensemble classifier is called a “weak learner.” The number of weak learners is denoted by 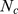. The training data were divided into 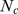 partitions using overlapped partitioning. A dimension reduction method was applied to these partitioned data, and then 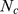 LDA weak learners were trained. The test data corresponding to a letter were processed to compute the scores, and then the scores were translated into a predicted letter. To evaluate the classification performance using thousands of training data, the proposed 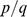 cross-validation was applied.

#### *p*/*q* cross-validation


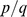
 cross-validation is a special cross-validation that can reduce the amount of training data. For a fair comparison of the classification accuracy, the amount of training data used in the offline analysis should be reduced to less than 1000. The traditional cross-validation method is not suitable because it provides at least 4500 training data in this case. Instead, we employed the proposed 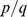 cross-validation that performed 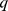-fold cross-validation, where 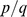 of all data were assigned to the training data.

First, the ERP training data are divided into 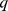 groups. Second, assuming that the groups are aligned around a circle, 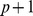 groups from 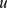
th group (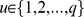) are sequentially selected. Then, 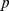 consecutive groups are assigned to the training data, and the last single group was assigned to the test data. The above procedures are repeated for all 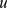. In total, 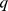 pairs of training and test data are prepared. For each pair, classification is performed. The classification accuracy can be computed as 
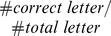
, where 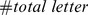 is the total number of letters and 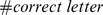 is the number of correct predictions among all pairs. It should be noted that 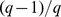 cross-validation is equivalent to the conventional 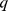-fold cross-validation.

**Figure 4:**
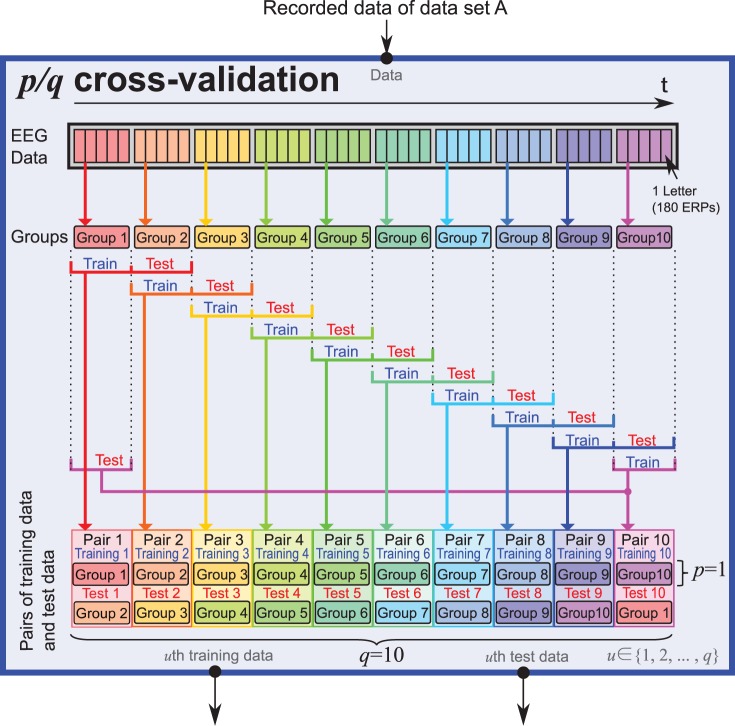
Procedure of 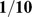 cross-validation used for the evaluation on data set A. In this case, 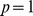 and 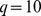. ERP data sets corresponding to fifty letters inputted by a participant were measured. The square aligned at the top illustrates a data set that contained 180 ERP data, 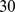 of which were labeled as target ERPs, while the others were labeled as non-target ERPs. These data sets were sorted according to measured time. The data sets were divided into ten groups. Then, two successive groups were selected. The former group was assigned to training data and the latter to test data. Then, each weak learner in the ensemble classifier was learned on the assigned training data and tested using the following test data.

In the present study, we have evaluated data set A using the 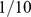 cross-validation as shown in Figure 4. In other words, five letters out of 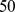 were assigned to the training data, which contained 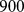 ERPs (9000 ERPs × 1/10). It takes 180.125 seconds to spell out five letters in the conditions of this online experiment, which does not overly tire the participant. In addition to the 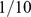 cross-validation, we also used the conventional 10-fold cross-validation (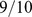 cross-validation) in order to compare the ensemble classifiers when a large amount of training data were provided. Thus, ERPs for 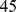 letters out of 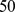 were used as training data, which contained 8100 ERPs (9000 ERPs × 9/10). The 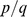 cross-validation was not applied to data set B because the competition data set has separated training and test data.

#### Overlapped partitioning

In a BCI study on ensemble classifiers, naive partitioning was used [23]. According to their use of naive partitioning, the given training data were divided into partitions by letters without overlaps. Due to the partitioning without overlaps, the amount of training data in a partition becomes small so that covariance matrices might not be estimated precisely. Instead of this method, we proposed a generalized partitioning method.

**Figure 5:**
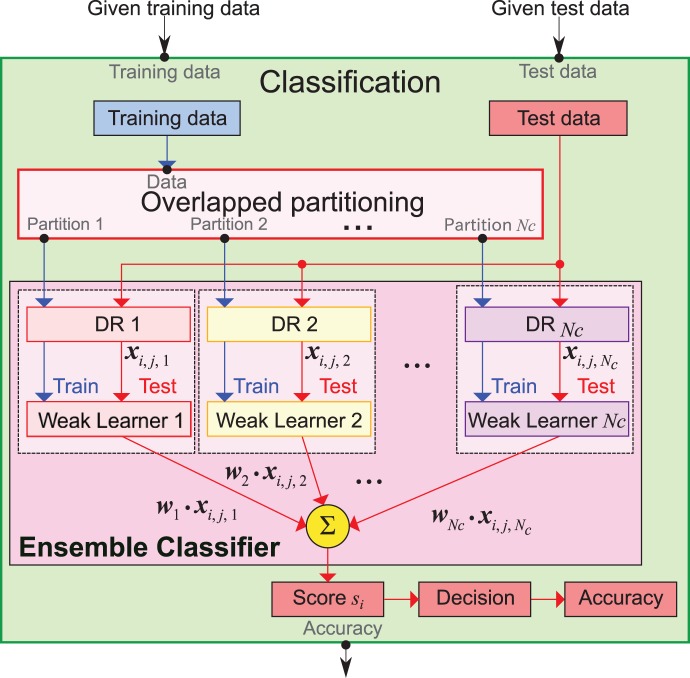
Training and testing procedure of the ensemble classifiers for P300-based BCI. Training data flows are represented by blue lines and test data flows are illustrated by red lines. The training data are divided into 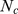 overlapped partitions (see Figure 6). One of three conditions for dimension reduction (DR) is applied to each partitioned data : the stepwise method, PCA, or none. Then, 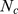 LDA weak learners are trained on these dimension-reduced data. The training data are used only for the training of weak learners as illustrated by blue lines. After the training session, the test data are processed to compute scores for decision making.

**Figure 6:**
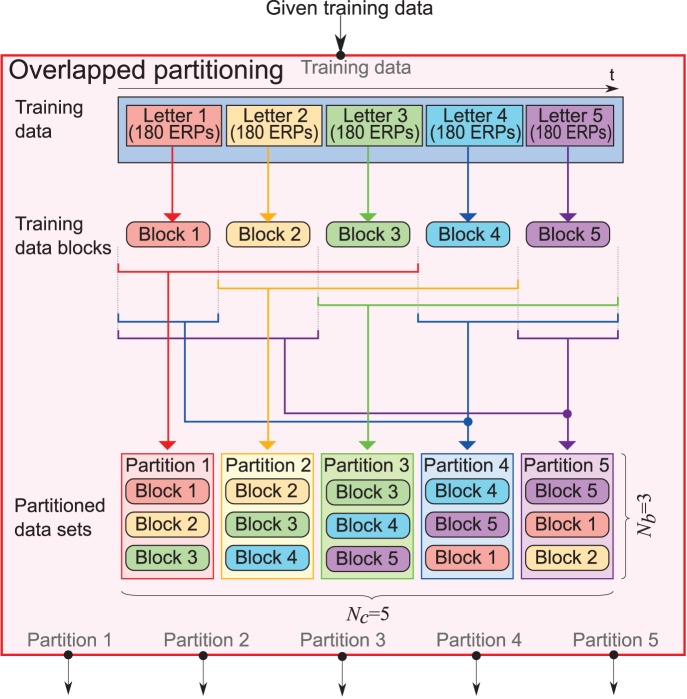
Overlapped partitioning when 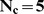 and 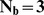. Training data were first divided into five blocks. Assuming that those five blocks were aligned around a circle, three continuous blocks were selected to form a partition. As a result, five partitions were prepared. The partitioned training data sets were used to train weak learners in the ensemble classifier.

All the procedures for training and testing the proposed ensemble classifier for P300-based BCI are shown in Figure 5. In overlapped partitioning, sets of training data are divided into 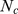 partitions, where the overlap of each partition is allowed. In the first step of the overlapped partitioning method, training data assigned to input commands were sorted by recorded time and were divided into 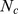 blocks without overlaps. Then, assuming that the blocks were aligned around a circle, 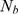 consecutive blocks from 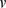
th block (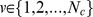) were selected to form a partition. The procedure was repeated for all 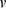. An example of overlapped partitioning is shown in Figure 6. Each weak learner was trained on the partitioned data (see Figure 5). The advantage of this partitioning method as compared to naive partitioning is that a larger amount of data are stored in each partition. Thus, overlapped partitioning may be robust against shortage of training data. In the present study, 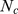 was fixed; however, 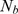 was varied in the offline analysis.

An ensemble classifier with the overlapped partitioning can be considered as a special case of bagging used in pattern recognition [37]. In the bagging, random sampling from available training data allowing overlap is used, which is also referred to as bootstrap sampling. In contrast, the overlapped partitioning does not have any randomness so that no duplicated partition is made except for a special case. Unlike a standard pattern recognition problem, a set of EEG was recorded for every letter, where 30 ERPs contained P300 and the other 150 ERPs did not. The random sampling out of the full set of EEG data runs the risk that only a few ERP data that contain P300 could be selected in a partition, which may deteriorate classification performance. Also the random sampling out of five blocks of EEG data is not effective because duplicated partitions could be prepared. The proposed overlapped partitioning does not have such risks and provides different partitions with a constant ratio of EEG data with P300 to those without it. Thus, the weak learners of the ensemble classifier can efficiently be trained by the overlapped partitioning.

#### Dimension reduction

A dimension reduction method has often been applied to the BCI because EEG data are usually high dimensional. However, the influences of the dimension reduction methods have not been evaluated for ensemble classifiers. In this study, one of three conditions for dimension reduction was applied: 2 dimension reduction methods (the stepwise method and PCA) and a control condition without dimension reduction (none).

- **Stepwise method** The stepwise method selects suitable spatiotemporal predictor variables for classification by forward and backward steps. First, an empty linear regression model is prepared, then variables are appended through the following steps. In the forward step, a variable is appended to the model, then the model was evaluated by an F-test. Through the F-test, p-value was computed, which is the probability of the occurrence of a result by chance. The variable is added if the p-value of the F-test is higher than a threshold 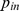. The forward step is repeated until no variable is appended. In the following backward step, a variable of the temporal model is removed and the model was also evaluated by the F-test. Then, the variable is removed if the p-value of the F-test is lower than a threshold 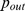. The backward step is continued until no variable is removed. Then, the forward step is repeated again. The final model is determined when no variable is appended to or removed from the model. The remaining variables in the final model are used for classification. More details are given in [21], [38]. We set 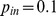 and 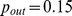, which were commonly used for P300-based BCI [21], [22].
- **Principal component analysis** The principal component analysis (PCA) is a typical dimension reduction method which is based on the eigenvalue decomposition [39], and has also been applied to P300-based BCI [10], [40]. In summary, the covariance matrix of training data is computed and then the eigenvalue decomposition is performed. The projected data using a normalized eigenvector corresponding to the largest eigenvalue is called the first principal component (PC). The other PCs can be calculated as well. We applied PCA to data in each partition, and then used 1–140 PCs for classification on data set A, 1–400 PCs for classification on data set B.

#### Linear discriminant analysis

Linear discriminant analysis (LDA) is a frequently used classifier for P300-based BCI. In the ensemble classifier, 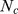 LDA weak learners are implemented. One of three conditions for dimension reduction is applied to the 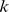
th partitioned data, and then the weight vector of the 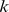
th LDA weak learner is trained as follows:
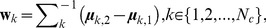
(1)where 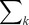 is a total covariance matrix over the target and non-target training data, and 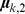 and 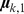 are the mean vectors of the target and non-target training data in the 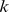
th partition. The trained weight vectors of each LDA weak learner are used to compute the score for the decision making. See [22] for more details of a single LDA classifier.

#### Decision making

To predict a letter, its corresponding test data were processed to compute scores for decision making. A test feature vector that belonged to the 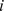
th intensification in the 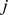
th sequence in the 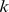
th partition after applying dimension reduction was denoted by 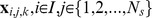. The score 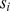 corresponding to an intensification was computed as.
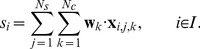
(2)


In the offline analysis, the number of intensification sequences 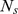 was varied from 1 to 15. The inputted letters were then predicted by finding maximum scores from row and column intensifications, respectively:
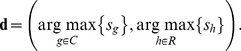
(3)


The first element of 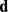 represents the column number of a predicted letter, while the second represents the row number. For example, 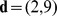 denotes “N” in Figure 3.

#### Special cases of overlapped partitioning

The ensemble classifiers with the proposed overlapped partitioning are equivalent to ensemble classifiers with naive partitioning or a single classifier in a special case. That is, the ensemble classifier with overlapped partitioning becomes the ensemble classifier with naive partitioning when 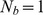 and 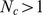. In this case, partitions do not overlap each other, which can be easily seen in Figure 6. Moreover, the ensemble classifier also behaves as a single classifier when 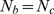. The scores in Equation 3 can be multiplied by an arbitrary 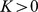:
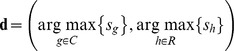


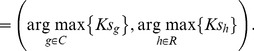
(4)


When 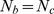, all the partitioned data sets are just duplications of all the given training data. After a dimension reduction method has been applied, the same data are stored in all partitions. As a result, all the weight vectors of the classifiers become the same:
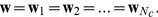
(5)


Since the final model of the stepwise method or the projection of the PCA is adjusted by the same training data, the test data after the dimension reduction should be the same:
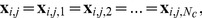
(6)


Considering Equation 5 and 6, the score for decision making instead of Equation 2 is computed by
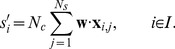
(7)


On the other hand, the score for a single classifier is formed as
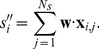
(8)


Thus, the relationship between the single classifier and the overlapped ensemble classifiers that have 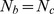 is
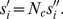
(9)


From Equation 4, 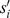 and 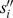 work in the same way for decision making. Therefore, the ensemble classifier with overlapped partitioning that satisfies 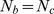 corresponds to a single classifier.

### Comparison Protocol

We evaluated varieties of ensemble classifiers with overlapped partitioning in order to ensure the influence of the degree of overlap together with dimension reduction methods. One of three different conditions for dimension reduction was applied: stepwise, PCA, or none. They are denoted by overlapped ensemble SWLDA (OSWLDA), overlapped ensemble PCA LDA (OPCALDA), and overlapped ensemble LDA (OLDA) classifiers, respectively.

Those 3 classifiers were evaluated on data sets A and B. Data set A, recorded by us, was analyzed in the small training data case using 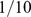 cross-validation and in the large training data case using 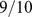 cross-validation (conventional 10-fold cross-validation). Thus, the same amount of the training data was provided for each ensemble classifier 900 training data for the former and 8100 training data for the latter. Additionally, in the cross-validation, the training and test data were clearly separated so that none of the training data were used as the test data. Data set B (BCI competition III data set II) was also analyzed using limited training data (ERPs corresponding to the first 5 letters) and using full training data (ERPs corresponding to 85 letters). The former contained 900 ERPs while the latter contained 15300 ERPs for training.

To confirm the influence of overlapped partitioning, the degree of overlaps 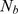 was varied, while the number of weak learners 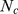 was fixed in the offline analysis. Evaluated combinations of 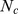 and 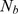 for data sets A and B were summarized in Tables 2 and 3, respectively. In particular, in the case 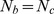, the ensemble classifier with overlapped partitioning is equivalent to the single classifier. In addition, in the case where 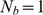 and 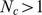, it behaves as a conventional ensemble classifier with naive partitioning. It should be noted that the algorithms were learned on 900 training data of both data sets, which was much smaller than the training data used in previous studies, for example, the 15300 training data used in the BCI competition III data set II [23], and 7560 data used in the BCI competition II data set IIb [41]. In our comparison, the single SWLDA which is commonly used in this field and the ensemble SWLDA proposed by Johnson *et al.* were compared.

**Table 2:** Evaluation parameters of ensemble classifiers with overlapped partitioning on data set A.

Evaluationmethod	#training letters	#test letters	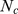	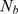	#training data for aweak learner (ERPs)
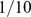 cross-valdiation	5 letters(900 ERPs)	50 letters	55555	12345	180360540720900
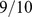 cross-valdiation(conventional10-fold cross-validation)	45 letters(8100 ERPs)	50 letters	45454545454545454545	151015202530354045	18090018002700360045005400630072008100

The data set A was evaluated by 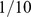 cross-validation (900 training data ) and 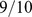 cross-validation (8100 training data). The number of weak learners 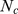 and the number of blocks 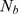 were the parameters of the overlapped partitioning. These evaluation methods and parameters determine the amount of training data for a weak learner in an ensemble classifier. The number of training letters (#training letters) is decided by 50 entire letters × 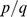. The number of training data for a weak learner (#training data for a weak learner) can be computed by 9000 entire ERPs × 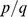 × 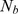/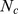.

**Table 3:** Evaluation parameters of ensemble classifiers with overlapped partitioning on data set B (BCI competition III data set II).

Evaluationmethod	#training letters	#test letters	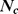	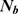	#training data for aweak learner (ERPs)
Limited training data(first 5 letters)	5 letters (900 ERPs)	100 letters	55555	12345	180360540720900
Full training data	85 letters	100 letters	1717171717171717171717171717171717	1234567891011121314151617	9001800270036004500540063007200810090009900108001170012600135001440015300

The ensemble classifiers were trained on limited training data (900 training data ) or full training data (15300 training data). The number of weak learners 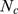 and the number of blocks 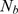 were parameters used in the overlapped partitioning. These evaluation methods and the parameters determine the amount of training data for a weak learner in an ensemble classifier. The number of training data for a weak learner (#training data for a weak learner) can be computed by given training ERPs × 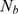/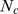.

For the statistical analysis of data set A, the effects of the intensification sequence (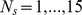), dimension reduction condition (stepwise, PCA or none), and degree of overlaps (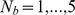) were evaluated by three-way repeated-measures ANOVA followed by post hoc pairwise t-tests with Bonferroni's method. No statistical analysis was applied to data set B because of the limited number of participants.

## Results

The classification performances of OSWLDA, OPCALDA, and OLDA were evaluated on data set A using 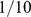 or 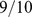 cross-validation and data set B with limited or full training data. The degree of overlap used in the overlapped partitioning (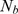) was varied while the number of weak learners in the ensemble classifier (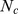) was fixed. As mentioned above, an overlapped ensemble classifier behaves as an ensemble classifier with naive partitioning when 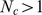 and 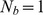, and becomes a single classifier when 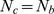.

### Data Set A Using 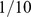 Cross-validation

**Figure 7:**
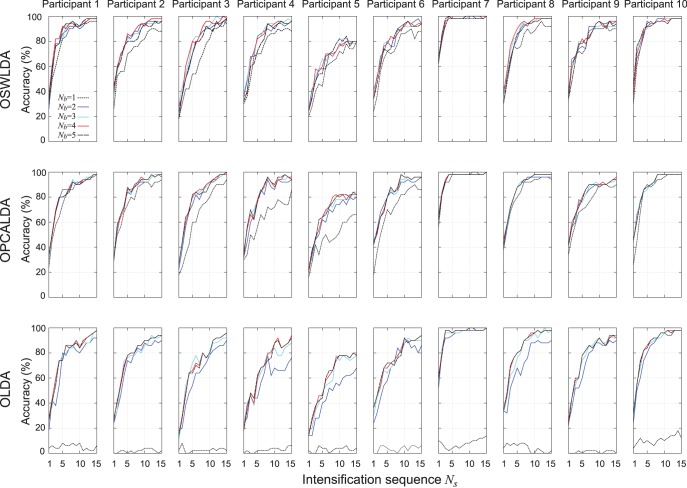
Classification performances of ensemble classifiers on data set A using 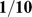 cross-validation. OSWLDA, OPCALDA and OLDA were trained on 900 ERPs. The influence of overlapped partitioning were evaluated by changing the degree of overlaps (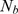) and the number of intensifications (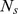). The classification performances of all participants were presented.

**Figure 8:**
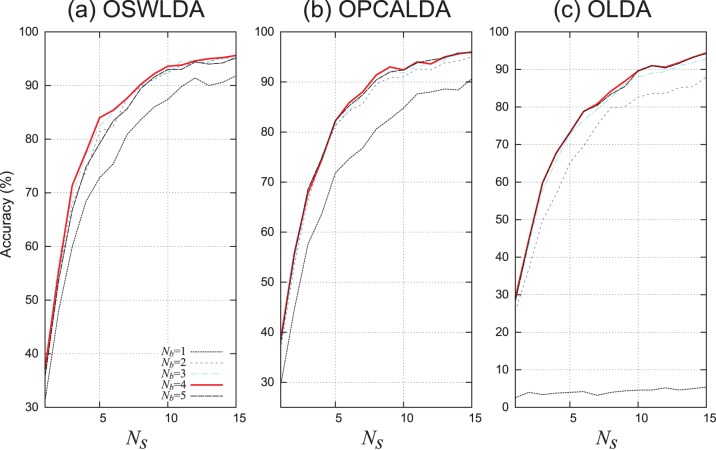
Mean classification performances of ensemble classifiers on data set A using 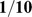 cross-validation. OSWLDA, OPCALDA and OLDA were trained on 900 ERPs. The classification accuracies were averaged over ten participants.

EEG data in data set A were classified by OSWLDA, OPCALDA, and OLDA using 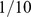 cross-validation using parameters in Table 2. The classification performances of these classifiers for each participant are shown in Figure 7. The mean accuracies of these algorithms are shown in Figure 8 and in Table 4.

**Table 4:** Mean classification accuracies (%) of OSWLDA, OPCALDA, and OLDA evaluated on data set A using 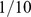 cross-validation.

Algorithms	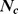	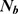	Intensification sequences 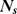														
			1	2	3	4	5	6	7	8	9	10	11	12	13	14	15
OSWLDA	5	1	31.2	48.0	60.0	68.4	72.8	75.4	80.8	83.6	86.0	87.4	89.8	91.4	90.0	90.6	91.8
		2	**37.6**	54.8	68.0	74.0	81.4	82.4	87.6	89.8	91.2	92.4	93.8	94.2	94.4	95.2	95.0
		3	36.4	**57.4**	69.4	77.2	82.4	85.0	**87.8**	90.0	91.4	92.6	**94.6**	**94.6**	94.6	95.0	94.8
		4	37.2	55.4	**71.4**	**77.6**	**84.0**	**85.4**	87.6	**90.2**	**92.2**	**93.6**	93.8	**94.6**	**95.0**	**95.2**	**95.6**
		5	35.6	53.6	66.8	74.8	79.2	83.4	85.6	89.4	91.6	93.0	93.0	94.4	94.0	94.2	95.2
OPCALDA	5	1	29.4	44.6	57.6	63.6	71.8	74.6	76.8	80.6	82.6	84.8	87.6	88.0	88.6	88.4	90.6
		2	36.6	53.4	66.4	75.0	81.2	84.2	85.6	89.6	90.8	91.0	92.6	92.4	93.8	94.2	95.0
		3	38.2	54.4	67.6	**75.2**	81.6	84.2	87.4	90.4	92.0	91.8	93.2	93.6	94.4	95.6	**96.0**
		4	**38.6**	**55.8**	67.8	74.4	**82.2**	**85.8**	**88.0**	**91.4**	**93.0**	**92.4**	**94.0**	93.6	**95.0**	95.6	**96.0**
		5	37.8	55.4	**68.6**	74.8	**82.2**	85.2	87.4	90.4	92.0	**92.4**	93.8	**94.4**	94.8	**95.8**	95.8
OLDA	5	1	2.6	4.0	3.4	3.8	4.0	4.2	3.2	4.0	4.4	4.6	4.6	5.2	4.6	5.0	5.4
		2	25.6	37.0	49.8	56.8	65.2	69.6	75.2	79.8	80.0	82.6	83.6	83.6	85.2	85.4	88.0
		3	28.2	43.2	57.8	65.8	72.8	76.4	79.4	82.4	86.2	88.0	89.0	89.4	91.0	91.8	92.8
		4	**29.2**	**44.8**	**59.8**	**67.8**	**73.2**	**78.8**	**80.8**	**84.2**	**86.8**	**89.6**	**91.0**	**90.6**	**91.8**	**93.2**	**94.4**
		5	28.2	44.4	**59.8**	67.6	**73.2**	**78.8**	80.4	83.4	85.4	**89.6**	**91.0**	90.4	91.6	**93.2**	94.2

The best accuracy among all 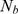 for each algorithm and each repetition is written in bold and the worst is underlined. An overlapped ensemble classifier becomes an ensemble classifier with naive partitioning when 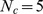 and 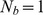. The classifier is equivalent to a single classifier when 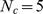 and 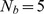.

The key finding was that OSWLDA showed higher classification performance than the single SWLDA classifier (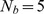) and ensemble SWLDA classifier with naive partitioning (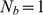) when 900 training data were provided. As can be seen in Table 4, most algorithms achieved the best performance when 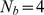, while the worst accuracy was observed when 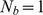. Regarding OLDA, when 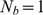, the classification accuracy was close to the chance level (1/36). As can be seen in Figure 8, OSWLDA (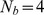) achieved a higher classification accuracy than the single SWLDA classifier (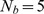), especially in 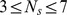. At 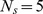, OSWLDA (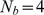) obtained an 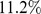 higher accuracy than the ensemble SWLDA classifier with naive partitioning and a 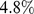 higher accuracy than the single SWLDA classifier. Moreover, OPCALDA (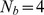) achieved a better classification accuracies than OPCALDA (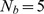) when 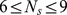, although the differences were small. In contrast, the accuracy of OLDA (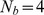) was close to that of the single LDA classifier (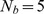), although OLDA (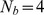) achieved slightly higher accuracies in some sequences.

A three-way repeated-measures ANOVA with the intensification sequence, dimension reduction conditions, and degree of overlap was applied. The main effects of the intensification sequence (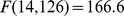, 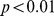), dimension reduction conditions (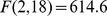, 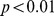), degree of overlap (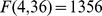, 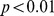) and all their interactions (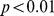 for all) were significant. In addition, significant differences between the dimension reduction conditions (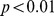 for all), and between pairs of 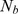, except for the pair 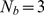 and 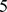 (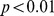 for all), were revealed by the post hoc pairwise t-test with Bonferroni's method.

### Data Set A Using 9/10 Cross-validation

**Figure 9:**
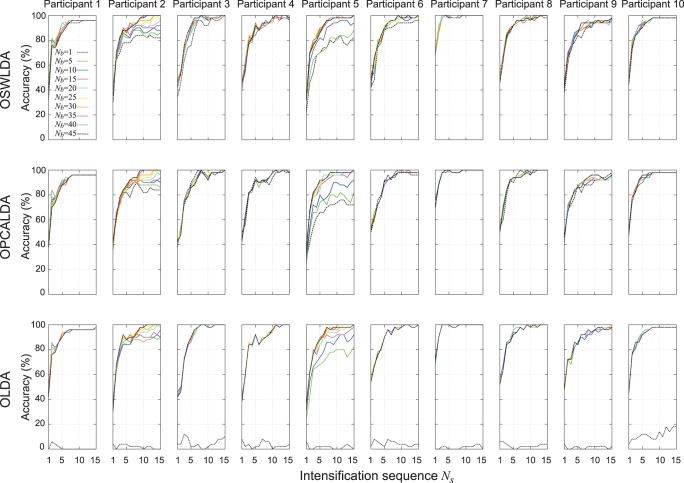
Classification performances of ensemble classifiers on data set A using 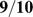 cross-validation. OSWLDA, OPCALDA and OLDA were trained on 8100 ERPs. Then the data set A was classified by those classifiers, changing 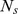 and 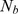. The classification performances of all participants were displayed.

**Figure 10:**
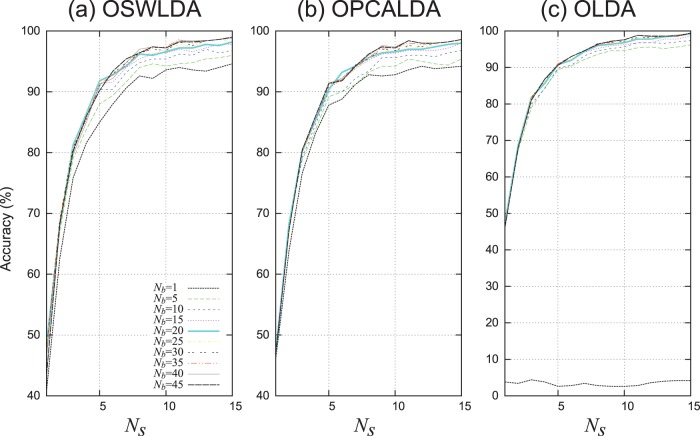
Mean classification performances of ensemble classifiers on data set A using 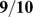 cross-validation. OSWLDA, OPCALDA and OLDA were trained on 8100 ERPs. The mean classification accuracies over ten participants were presented.

EEG data in data set A were also classified by the three algorithms using 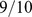 cross-validation using parameters in Table 2. Classification performances of the three algorithms for each individual participant are shown in Figure 9. The mean classification performances are shown in Figure 10 and Table 5.

**Table 5:** Mean classification accuracies (%) of OSWLDA, OPCALDA, and OLDA evaluated on data set A using 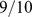 cross-validation.

Algorithms	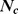	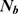	Intensification sequences 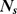														
			1	2	3	4	5	6	7	8	9	10	11	12	13	14	15
OSWLDA	45	1	40.8	62.6	75.8	81.6	85.0	88.0	90.6	92.6	92.2	93.6	94.0	93.6	93.4	94.0	94.6
		5	47.4	67.0	79.0	83.8	88.0	89.4	91.6	94.0	94.6	94.2	94.6	94.8	95.4	95.6	96.0
		10	47.2	68.2	80.4	86.2	90.4	90.4	93.2	94.8	95.4	95.4	96.2	96.0	97.0	96.4	96.8
		15	47.8	68.6	80.6	85.4	91.4	91.6	94.0	95.4	96.2	96.4	97.0	96.6	97.2	97.8	97.8
		20	**48.4**	68.0	**81.2**	**86.4**	**91.8**	92.8	94.2	96.2	96.0	96.6	97.2	97.2	97.8	97.6	98.2
		25	47.8	69.0	81.0	86.2	91.6	92.8	94.6	96.6	96.2	97.0	97.8	97.4	**98.4**	**98.6**	98.2
		30	47.8	**69.2**	80.8	85.6	91.0	93.0	94.8	96.4	97.0	96.8	98.2	97.6	**98.4**	**98.6**	98.8
		35	47.4	68.8	80.0	85.8	91.2	92.8	94.4	96.4	97.2	**97.4**	**98.6**	98.0	**98.4**	**98.6**	98.8
		40	46.2	68.0	79.6	85.0	91.2	92.2	94.8	**97.0**	**97.4**	97.2	98.4	**98.4**	**98.4**	**98.6**	98.8
		45	43.4	68.2	80.2	86.0	90.2	**93.4**	**95.4**	96.4	**97.4**	97.2	98.2	98.2	**98.4**	**98.6**	**99.0**
OPCALDA	45	1	46.0	63.8	76.6	83.2	87.8	88.8	91.2	92.8	92.6	92.8	93.6	94.2	93.8	94.0	94.2
		5	46.0	**68.8**	78.8	83.8	89.2	90.0	91.2	93.2	94.2	94.2	95.4	95.0	94.6	94.4	95.4
		10	46.4	67.6	79.2	84.6	90.0	90.0	92.2	93.4	95.6	95.6	96.0	96.0	95.8	96.4	96.8
		15	47.4	68.2	80.0	**86.0**	90.6	92.0	93.8	95.2	96.2	96.4	96.8	96.8	96.8	97.2	98.0
		20	47.0	68.2	80.2	85.4	90.4	**93.2**	94.2	95.6	96.4	96.6	97.0	97.0	97.4	97.8	98.0
		25	47.0	66.6	80.2	85.4	91.0	92.4	93.8	95.6	96.6	97.0	97.4	97.2	97.8	**98.2**	98.2
		30	**47.4**	67.0	**80.6**	85.4	91.0	92.2	**94.4**	95.4	97.0	96.8	97.6	97.4	**98.0**	**98.2**	**98.6**
		35	47.0	67.4	80.2	85.4	91.2	92.0	94.0	95.6	97.2	97.2	98.0	97.8	**98.0**	**98.2**	**98.6**
		40	46.6	67.0	**80.6**	85.4	**91.4**	92.2	94.2	96.0	**97.6**	**97.4**	**98.4**	**98.0**	**98.0**	**98.2**	**98.6**
		45	46.8	67.2	80.4	**86.0**	**91.4**	91.8	94.2	**96.2**	97.4	97.2	**98.4**	**98.0**	**98.0**	**98.2**	**98.6**
OLDA	45	1	3.8	3.4	4.4	3.8	2.6	2.8	3.4	2.8	2.6	2.6	2.8	3.6	4.0	4.2	4.2
		5	46.6	67.2	79.0	84.4	89.4	90.4	92.2	93.6	94.6	94.6	95.4	95.6	95.2	95.6	96.2
		10	**47.8**	68.4	80.0	84.2	89.8	90.6	93.2	94.6	95.2	95.6	96.8	96.8	96.6	97.0	97.4
		15	47.6	**68.6**	81.8	85.4	90.6	91.8	94.2	95.2	96.0	96.2	96.8	96.8	97.8	97.8	98.6
		20	47.2	**68.6**	81.8	85.6	90.8	92.0	94.4	96.0	96.4	96.8	97.8	97.8	98.4	**98.8**	99.2
		25	46.8	68.2	**82.0**	86.2	**91.2**	92.2	**94.8**	96.4	96.6	97.0	98.0	97.8	**98.6**	**98.8**	**99.6**
		30	46.4	67.4	81.8	86.4	91.0	**93.0**	94.6	**96.6**	96.8	97.2	98.2	98.2	**98.6**	**98.8**	99.4
		35	46.4	68.0	81.6	**86.8**	91.0	**93.0**	94.6	96.2	97.0	**97.6**	**98.8**	98.4	**98.6**	**98.8**	99.4
		40	46.0	68.0	81.4	**86.8**	90.8	**93.0**	94.6	96.4	**97.2**	**97.6**	**98.8**	98.4	**98.6**	98.6	99.4
		45	46.0	68.0	81.0	**86.8**	90.6	**93.0**	94.6	96.4	**97.2**	**97.6**	**98.8**	**98.6**	**98.6**	98.6	99.4

The best accuracy among all 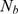 for each algorithm and each repetition is written in bold and the worst is underlined. An overlapped ensemble classifier becomes an ensemble classifier with naive partitioning when 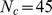 and 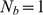. The classifier is equivalent to a single classifier when 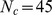 and 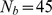.

The classification performance of ensemble classifiers with the overlapped partitioning were as well as, or slightly better than that of the single classifier when 8100 training data were provided. As shown in Figure 10, the worst classification performance was achieved by the ensemble classifiers (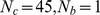) for all algorithms, which was the same as the analysis of data set A using 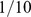 cross-validation. However, only a little performance improvement of the overlapped ensemble classifiers can be found when compared to the single classifier (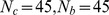).

A three-way repeated-measures ANOVA with the intensification sequence, dimension reduction conditions, and degree of overlap was applied. The main effects of the intensification sequence (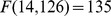, 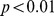), dimension reduction conditions (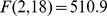, 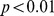), degree of overlap (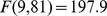, 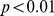) and all their interactions (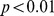 for all) were significant. In addition the post hoc pairwise t-test was applied. Significant differences between the dimension reduction conditions (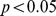 for all) were revealed. Also, significant differences between the pairs containing 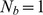, 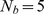, 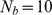, and 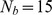 (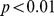 for all) were revealed.

### Data Set B with Limited Training Data

EEG data in data set B were classified by OSWLDA, OPCALDA and OLDA using 900 training data using parameters in Table 3. Classification performances of OSWLDA, OPCALDA, and OLDA evaluated on data set B using a limited amount of training data (900 ERPs) are shown in Tables 6, 7, and 8, respectively.

**Table 6:** Classification accuracies (%) of OSWLDA on data set B with limited training data.

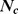	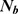	Participants	Intensification sequences 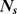															
			1	2	3	4	5	6	7	8	9	10	11	12	13	14	15	
5	1	A	5	3	7	8	9	11	6	7	7	10	8	10	10	7	11	
		B	5	4	8	9	9	8	7	8	5	9	9	9	11	15	13	
		Mean	5.0	3.5	7.5	8.5	9.0	9.5	6.5	7.5	6.0	9.5	8.5	9.5	10.5	11.0	12.0	
5	2	A	6	2	6	9	7	4	6	10	7	9	10	10	10	13	10	
		B	3	3	3	7	5	5	1	2	1	3	3	1	0	1	0	
		Mean	4.5	2.5	4.5	8.0	6.0	4.5	3.5	6.0	4.0	6.0	6.5	5.5	5.0	7.0	5.0	
5	3	A	14	17	24	30	24	33	36	37	40	47	54	56	61	65	67	
		B	20	23	34	40	49	51	53	49	59	61	65	67	67	72	74	
		Mean	**17.0**	20.0	29.0	35.0	36.5	42.0	44.5	43.0	49.5	54.0	59.5	61.5	64.0	68.5	70.5	
5	4	A	10	24	21	32	28	36	39	43	51	53	57	59	64	63	67	
		B	18	28	41	44	60	67	62	63	66	68	74	79	81	80	82	
		Mean	14.0	**26.0**	**31.0**	**38.0**	**44.0**	**51.5**	**50.5**	**53.0**	**58.5**	**60.5**	**65.5**	**69.0**	**72.5**	**71.5**	**74.5**	
5	5	A	3	14	20	26	23	24	31	38	41	53	50	57	53	62	68	
		B	15	24	36	37	50	49	50	48	49	56	58	61	66	70	71	
		Mean	9.0	19.0	28.0	31.5	36.5	36.5	40.5	43.0	45.0	54.5	54.0	59.0	59.5	66.0	69.5	

The best mean accuracy among all 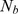 for each repetition is written in bold and the worst is underlined. An overlapped ensemble classifier becomes an ensemble classifier with naive partitioning when 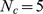 and 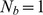. The classifier is equivalent to a single classifier when 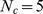 and 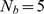.

**Table 7:** Classification accuracies (%) of OPCALDA on data set B with limited training data.

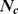	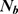	Participants	Intensification sequences 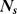														
			1	2	3	4	5	6	7	8	9	10	11	12	13	14	15
5	1	A	4	7	6	5	6	6	10	8	6	7	4	6	6	4	4
		B	4	7	2	4	4	5	3	3	4	6	5	5	4	4	3
		Mean	4.0	7.0	4.0	4.5	5.0	5.5	6.5	5.5	5.0	6.5	4.5	5.5	5.0	4.0	3.5
5	2	A	0	2	1	1	3	3	3	1	4	4	4	3	3	4	4
		B	7	4	1	2	4	3	4	4	6	5	4	4	2	2	2
		Mean	3.5	3.0	1.0	1.5	3.5	3.0	3.5	2.5	5.0	4.5	4.0	3.5	2.5	3.0	3.0
5	3	A	12	17	22	22	31	36	37	41	47	57	55	60	59	60	65
		B	10	24	29	34	39	34	41	39	46	48	53	54	59	61	61
		Mean	**11.0**	20.5	**25.5**	**28.0**	**35.0**	**35.0**	39.0	40.0	46.5	**52.5**	**54.0**	57.0	59.0	60.5	63.0
5	4	A	7	17	17	19	30	31	42	41	48	53	56	57	57	62	65
		B	11	26	27	33	40	39	46	49	49	49	51	59	63	64	62
		Mean	9.0	**21.5**	22.0	26.0	**35.0**	**35.0**	**44.0**	**45.0**	**48.5**	51.0	53.5	**58.0**	**60.0**	**63.0**	**63.5**
5	5	A	7	16	17	18	26	27	36	38	43	48	49	53	53	56	60
		B	7	23	25	32	42	40	45	44	50	47	48	52	60	63	60
		Mean	7.0	19.5	21.0	25.0	34.0	33.5	40.5	41.0	46.5	47.5	48.5	52.5	56.5	59.5	60.0

The best mean accuracy among all 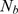 for each repetition is written in bold and the worst is underlined. An overlapped ensemble classifier becomes an ensemble classifier with naive partitioning when 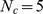 and 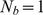. The classifier is equivalent to a single classifier when 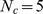 and 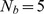.

**Table 8:** Classification accuracies (%) of OLDA on data set B with limited training data.

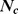	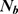	Participants	Intensification sequences 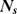														
			1	2	3	4	5	6	7	8	9	10	11	12	13	14	15
5	1	A	1	3	5	4	2	2	4	5	3	3	2	3	3	5	3
		B	6	3	2	3	4	6	6	4	6	4	4	4	8	7	8
		Mean	3.5	3.0	3.5	3.5	3.0	4.0	5.0	4.5	4.5	3.5	3.0	3.5	5.5	6.0	5.5
5	2	A	3	4	7	4	6	3	2	3	2	2	1	1	2	2	2
		B	2	7	6	6	4	6	10	11	7	8	8	10	11	9	11
		Mean	2.5	**5.5**	**6.5**	5.0	5.0	4.5	6.0	7.0	4.5	5.0	4.5	5.5	**6.5**	5.5	6.5
5	3	A	2	1	0	0	1	2	1	1	1	1	1	1	1	1	1
		B	2	3	5	5	4	2	1	4	3	5	3	3	1	1	2
		Mean	2.0	2.0	2.5	2.5	2.5	2.0	1.0	2.5	2.0	3.0	2.0	2.0	1.0	1.0	1.5
5	4	A	0	1	2	2	3	3	1	1	2	2	2	2	1	4	2
		B	2	1	2	2	3	2	3	4	4	4	4	3	3	5	4
		Mean	1.0	1.0	2.0	2.0	3.0	2.5	2.0	2.5	3.0	3.0	3.0	2.5	2.0	4.5	3.0
5	5	A	7	8	5	8	5	5	6	10	8	6	8	8	7	8	8
		B	4	3	4	7	7	6	8	9	11	9	8	8	5	8	6
		Mean	**5.5**	**5.5**	4.5	**7.5**	**6.0**	**5.5**	**7.0**	**9.5**	**9.5**	**7.5**	**8.0**	**8.0**	6.0	**8.0**	**7.0**

The best mean accuracy among all 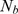 for each repetition is written in bold and the worst is underlined. An overlapped ensemble classifier becomes an ensemble classifier with naive partitioning when 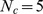 and 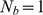. The classifier is equivalent to a single classifier when 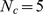 and 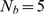.

The OSWLDA and OPCALDA (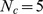 and 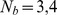) achieved better classification accuracies than those with naive partitioning (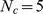 and 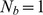) and the single classifiers (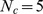 and 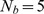) when 900 training data were available. As for OSWLDA, the best classification accuracies can be seen when 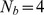. Further, most of the best mean classification performances of OPCALDA can be seen when 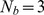 or 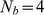. These tendencies are similar to the analysis of data set A using 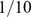 cross-validation. OSWLDA achieved about 10% (15% at best when 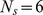) higher mean classification accuracy than the single SWLDA classifier (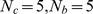). OPCALDA also achieved a 5.5% higher mean classification accuracy than the single PCALDA classifier (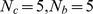) when 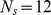. However, all of the classification performances of OLDA were close to chance level.

### Data Set B with Full Training Data

EEG data of data set B were classified by OSWLDA, OPCALDA and OLDA using 15300 training data using parameters in Table 3. Classification performances of the three algorithms evaluated on data set B using full training data (15300 ERPs) are presented in Tables 9, 10, and 11, respectively.

**Table 9:** Classification accuracies (%) of OSWLDA on data set B with full training data.

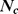	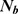	Participants	Intensification sequences 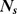														
			1	2	3	4	5	6	7	8	9	10	11	12	13	14	15
17	1	A	17	28	49	53	60	62	65	72	79	82	83	85	86	91	90
		B	45	62	66	70	77	84	87	89	91	93	94	97	96	97	96
		Mean	31.0	45.0	57.5	61.5	68.5	73.0	76.0	80.5	85.0	87.5	88.5	91.0	91.0	94.0	93.0
17	2	A	19	30	48	59	64	68	75	76	82	86	85	88	91	94	96
		B	46	63	67	70	79	87	90	92	91	94	94	97	97	98	98
		Mean	32.5	46.5	57.5	64.5	71.5	77.5	82.5	84	86.5	90	89.5	92.5	94	96	97.0
17	3	A	21	38	59	62	69	76	81	82	85	85	85	92	93	97	96
		B	49	64	69	73	81	86	87	91	93	95	94	95	96	97	97
		Mean	35.0	51.0	**64.0**	**67.5**	**75.0**	**81.0**	**84.0**	**86.5**	89.0	90.0	89.5	93.5	94.5	97.0	96.5
17	4	A	19	36	54	65	67	71	79	80	81	85	85	90	89	94	96
		B	51	64	71	70	80	85	86	91	93	95	94	95	96	94	97
		Mean	35.0	50.0	62.5	**67.5**	73.5	78.0	82.5	85.5	87.0	90.0	89.5	92.5	92.5	94.0	96.5
17	5	A	21	37	59	63	64	72	80	79	82	85	87	91	92	94	97
		B	49	64	69	71	78	86	87	92	93	95	94	96	96	96	97
		Mean	35.0	50.5	**64.0**	67.0	71.0	79.0	83.5	85.5	87.5	90.0	90.5	93.5	94.0	95.0	97.0
17	6	A	20	37	54	60	61	73	77	78	85	87	87	89	91	94	96
		B	46	63	69	70	82	86	88	92	94	95	95	95	96	95	97
		Mean	33.0	50.0	61.5	65.0	71.5	79.5	82.5	85.0	**89.5**	91.0	91.0	92.0	93.5	94.5	96.5
17	7	A	22	39	55	63	63	74	79	78	84	87	86	91	93	95	99
		B	48	63	70	70	81	87	88	92	94	95	94	95	96	96	97
		Mean	35.0	51.0	62.5	66.5	72.0	80.5	83.5	85.0	89.0	91.0	90.0	93.0	94.5	95.5	**98.0**
17	8	A	22	36	52	59	64	72	76	79	81	87	87	90	94	94	98
		B	46	68	69	70	81	88	89	92	94	95	95	95	95	95	97
		Mean	34.0	**52.0**	60.5	64.5	72.5	80.0	82.5	85.5	87.5	91.0	91.0	92.5	94.5	94.5	97.5
17	9	A	27	37	51	60	65	73	77	80	83	89	89	93	95	95	99
		B	45	64	69	69	79	87	89	92	94	95	95	95	95	95	97
		Mean	**36.0**	50.5	60.0	64.5	72.0	80.0	83.0	86.0	88.5	**92.0**	**92.0**	94.0	95.0	95.0	**98.0**
17	10	A	22	35	54	62	63	72	74	77	83	88	87	93	95	94	98
		B	48	66	70	69	80	85	91	92	93	95	94	95	96	96	97
		Mean	35.0	50.5	62.0	65.5	71.5	78.5	82.5	84.5	88.0	91.5	90.5	94.0	**95.5**	95.0	97.5
17	11	A	22	36	56	59	65	75	76	79	83	88	86	91	94	95	99
		B	44	66	71	70	80	87	91	92	94	94	94	95	96	95	97
		Mean	33.0	51.0	63.5	64.5	72.5	**81.0**	83.5	85.5	88.5	91.0	90.0	93.0	95.0	95.0	**98.0**
17	12	A	22	34	55	62	66	75	74	77	82	88	87	93	95	96	98
		B	43	67	71	72	83	86	91	92	94	95	95	96	95	96	97
		Mean	32.5	50.5	63.0	67.0	74.5	80.5	82.5	84.5	88.0	91.5	91.0	**94.5**	95.0	96.0	97.5
17	13	A	23	34	53	59	65	74	75	77	83	87	87	93	95	97	97
		B	42	63	69	70	81	86	92	92	94	94	94	95	95	96	97
		Mean	32.5	48.5	61.0	64.5	73.0	80.0	83.5	84.5	88.5	90.5	90.5	94.0	95.0	**96.5**	97.0
17	14	A	24	37	53	60	67	73	74	79	82	87	89	93	95	95	97
		B	43	65	69	71	82	86	91	92	93	95	94	95	95	95	97
		Mean	33.5	51.0	61.0	65.5	74.5	79.5	82.5	85.5	87.5	91.0	91.5	94.0	95.0	95.0	97.0
17	15	A	23	34	52	61	66	71	74	78	81	87	87	91	92	95	96
		B	44	62	69	72	81	86	92	92	94	95	94	95	95	95	97
		Mean	33.5	48.0	60.5	66.5	73.5	78.5	83.0	85.0	87.5	91.0	90.5	93.0	93.5	95.0	96.5
17	16	A	22	34	49	60	67	70	73	79	81	88	87	90	94	95	96
		B	45	65	69	72	83	87	93	92	94	95	94	95	95	95	97
		Mean	33.5	49.5	59.0	66.0	**75.0**	78.5	83.0	85.5	87.5	91.5	90.5	92.5	94.5	95.0	96.5
17	17	A	21	32	51	51	60	65	68	76	79	86	85	89	94	93	94
		B	42	62	69	70	82	84	88	91	92	95	94	94	94	94	97
		Mean	31.5	47.0	60.0	60.5	71.0	74.5	78.0	83.5	85.5	90.5	89.5	91.5	94.0	93.5	95.5

The best mean accuracy among all 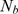 for each repetition is written in bold and the worst is underlined. An overlapped ensemble classifier becomes an ensemble classifier with naive partitioning when 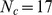 and 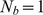. The classifier is equivalent to a single classifier when 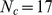 and 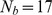.

**Table 10:** Classification accuracies (%) of OPCALDA on data set B with full training data.

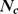	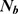	Participants	Intensification sequences 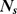														
			1	2	3	4	5	6	7	8	9	10	11	12	13	14	15
17	1	A	16	34	50	54	62	66	72	76	79	84	86	91	90	95	95
		B	39	57	64	73	80	86	89	91	91	94	93	94	93	94	94
		Mean	27.5	45.5	57.0	63.5	71.0	76.0	80.5	83.5	85.0	89.0	89.5	92.5	91.5	94.5	94.5
17	2	A	21	32	49	57	64	69	73	76	78	86	84	91	92	95	97
		B	43	63	69	75	81	85	89	91	90	93	95	96	94	95	96
		Mean	**32.0**	47.5	**59.0**	66.0	**72.5**	77.0	81.0	83.5	84.0	89.5	89.5	93.5	93.0	95.0	96.5
17	3	A	18	37	50	60	65	72	74	76	79	87	88	91	94	95	96
		B	44	62	63	75	79	86	89	92	90	94	94	98	96	96	97
		Mean	31.0	**49.5**	56.5	67.5	72.0	79.0	81.5	84.0	84.5	**90.5**	**91.0**	**94.5**	**95.0**	95.5	96.5
17	4	A	19	37	50	61	64	72	76	78	79	86	88	92	94	95	96
		B	42	62	62	77	78	85	88	90	90	94	94	96	95	97	97
		Mean	30.5	**49.5**	56.0	**69.0**	71.0	78.5	82.0	84.0	84.5	90.0	**91.0**	94.0	94.5	**96.0**	96.5
17	5	A	19	36	52	62	65	72	76	78	80	85	88	92	94	95	96
		B	42	62	63	75	78	85	88	90	90	94	94	97	95	96	97
		Mean	30.5	49.0	57.5	68.5	71.5	78.5	82.0	84.0	**85.0**	89.5	**91.0**	**94.5**	94.5	95.5	96.5
17	6	A	19	34	51	63	64	73	75	81	80	86	87	92	94	95	97
		B	43	60	62	75	78	85	88	90	90	94	94	96	95	97	97
		Mean	31.0	47.0	56.5	**69.0**	71.0	79.0	81.5	85.5	**85.0**	90.0	90.5	94.0	94.5	**96.0**	**97.0**
17	7	A	19	35	50	63	65	73	75	81	80	87	87	92	94	95	97
		B	43	61	62	75	79	86	89	91	89	94	94	95	95	96	96
		Mean	31.0	48.0	56.0	**69.0**	72.0	**79.5**	82.0	86.0	84.5	**90.5**	90.5	93.5	94.5	95.5	96.5
17	8	A	18	34	50	62	62	70	76	81	80	86	87	92	94	95	97
		B	44	61	61	75	79	86	89	90	89	94	93	95	94	96	96
		Mean	31.0	47.5	55.5	68.5	70.5	78.0	82.5	85.5	84.5	90.0	90.0	93.5	94.0	95.5	96.5
17	9	A	19	34	50	62	60	68	76	82	80	86	87	92	94	94	97
		B	44	61	61	74	79	86	89	90	89	94	93	95	94	95	96
		Mean	31.5	47.5	55.5	68.0	69.5	77.0	82.5	86.0	84.5	90.0	90.0	93.5	94.0	94.5	96.5
17	10	A	19	34	50	61	60	67	76	82	80	86	87	92	94	94	97
		B	44	62	61	74	80	87	89	91	89	93	93	95	93	94	96
		Mean	31.5	48.0	55.5	67.5	70.0	77.0	82.5	**86.5**	84.5	89.5	90.0	93.5	93.5	94.0	96.5
17	11	A	20	33	51	59	58	66	76	82	80	86	87	92	94	94	97
		B	44	61	61	73	81	87	89	91	89	93	93	95	93	95	96
		Mean	**32.0**	47.0	56.0	66.0	69.5	76.5	82.5	**86.5**	84.5	89.5	90.0	93.5	93.5	94.5	96.5
17	12	A	19	33	51	60	57	65	76	81	80	86	88	92	94	94	97
		B	44	61	61	73	81	87	90	91	89	93	93	95	93	95	96
		Mean	31.5	47.0	56.0	66.5	69.0	76.0	83.0	86.0	84.5	89.5	90.5	93.5	93.5	94.5	96.5
17	13	A	18	33	51	59	56	65	75	81	80	86	88	92	92	94	96
		B	44	62	61	73	81	88	90	90	89	93	93	95	93	95	97
		Mean	31.0	47.5	56.0	66.0	68.5	76.5	82.5	85.5	84.5	89.5	90.5	93.5	92.5	94.5	96.5
17	14	A	18	33	50	58	56	65	76	80	81	86	87	92	92	94	96
		B	44	62	61	73	81	88	91	90	89	93	93	94	93	95	97
		Mean	31.0	47.5	55.5	65.5	68.5	76.5	**83.5**	85.0	**85.0**	89.5	90.0	93.0	92.5	94.5	96.5
17	15	A	18	35	49	57	56	64	76	80	81	86	84	92	92	93	95
		B	44	63	61	73	81	90	91	90	89	93	93	95	93	95	97
		Mean	31.0	49.0	55.0	65.0	68.5	77.0	**83.5**	85.0	**85.0**	89.5	88.5	93.5	92.5	94.0	96.0
17	16	A	18	35	49	56	56	64	76	79	80	86	84	91	92	93	95
		B	44	63	62	73	81	90	91	90	90	93	93	94	93	95	97
		Mean	31.0	49.0	55.5	64.5	68.5	77.0	**83.5**	84.5	**85.0**	89.5	88.5	92.5	92.5	94.0	96.0
17	17	A	18	34	49	56	56	63	76	78	79	84	84	91	92	92	95
		B	46	64	62	73	81	90	91	90	90	93	93	93	93	95	97
		Mean	**32.0**	49.0	55.5	64.5	68.5	76.5	**83.5**	84.0	84.5	88.5	88.5	92.0	92.5	93.5	96.0

The best mean accuracy among all 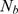 for each repetition is written in bold and the worst is underlined. An overlapped ensemble classifier becomes an ensemble classifier with naive partitioning when 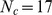 and 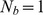. The classifier is equivalent to a single classifier when 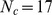 and 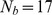.

**Table 11:** Classification accuracies (%) of OLDA on data set B with full training data.

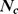	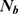	Participants	Intensification sequences 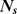														
			1	2	3	4	5	6	7	8	9	10	11	12	13	14	15
17	1	A	3	4	2	4	4	5	8	9	11	9	8	14	11	12	15
		B	0	4	4	1	0	1	0	1	4	0	2	2	3	3	3
		Mean	1.5	4.0	3.0	2.5	2.0	3.0	4.0	5.0	7.5	4.5	5.0	8.0	7.0	7.5	9.0
17	2	A	20	36	48	55	59	64	73	78	78	90	87	92	92	93	95
		B	35	52	63	63	69	78	82	81	83	88	91	93	89	91	95
		Mean	27.5	44.0	55.5	59.0	64.0	71.0	77.5	79.5	80.5	89.0	89.0	92.5	90.5	92.0	95.0
17	3	A	24	39	53	54	62	67	75	77	81	87	88	92	95	95	97
		B	43	65	67	74	78	85	87	87	88	94	94	94	93	93	94
		Mean	33.5	**52.0**	60.0	64.0	70.0	76.0	81.0	82.0	84.5	**90.5**	91.0	93.0	94.0	94.0	**95.5**
17	4	A	25	35	52	56	62	68	76	78	82	87	89	95	97	95	97
		B	43	67	67	75	79	85	87	89	89	94	94	94	93	93	94
		Mean	**34.0**	51.0	59.5	65.5	70.5	76.5	81.5	83.5	**85.5**	**90.5**	91.5	94.5	95.0	94.0	**95.5**
17	5	A	25	33	51	55	62	69	76	77	81	87	89	96	96	95	97
		B	43	68	70	75	79	84	87	89	89	94	95	94	94	95	93
		Mean	**34.0**	50.5	**60.5**	65.0	70.5	76.5	81.5	83.0	85.0	**90.5**	92.0	**95.0**	95.0	**95.0**	95.0
17	6	A	25	31	52	58	64	69	75	78	82	86	91	96	96	96	97
		B	43	67	69	75	79	84	88	89	89	94	95	94	94	94	93
		Mean	**34.0**	49.0	**60.5**	**66.5**	**71.5**	76.5	81.5	83.5	**85.5**	90.0	93.0	**95.0**	95.0	**95.0**	95.0
17	7	A	25	31	51	58	62	69	75	78	82	86	91	96	97	96	97
		B	41	67	70	73	79	85	88	90	89	94	95	94	94	94	93
		Mean	33.0	49.0	**60.5**	65.5	70.5	**77.0**	81.5	84.0	**85.5**	90.0	93.0	**95.0**	**95.5**	**95.0**	95.0
17	8	A	24	32	49	59	63	68	76	79	82	86	91	96	95	96	97
		B	40	67	71	73	79	84	88	90	88	94	95	94	94	94	93
		Mean	32.0	49.5	60.0	66.0	71.0	76.0	**82.0**	84.5	85.0	90.0	93.0	**95.0**	94.5	**95.0**	95.0
17	9	A	26	34	49	60	63	67	76	79	81	85	91	96	95	96	97
		B	41	66	71	73	79	85	87	90	88	93	95	94	94	93	93
		Mean	33.5	50.0	60.0	**66.5**	71.0	76.0	81.5	84.5	84.5	89.0	93.0	**95.0**	94.5	94.5	95.0
17	10	A	25	34	48	60	62	67	76	80	81	85	92	96	95	97	97
		B	40	65	70	71	79	86	87	90	88	93	95	94	94	93	94
		Mean	32.5	49.5	59.0	65.5	70.5	76.5	81.5	85.0	84.5	89.0	**93.5**	**95.0**	94.5	**95.0**	**95.5**
17	11	A	24	33	48	60	61	65	77	80	81	86	92	96	96	96	96
		B	40	65	69	71	79	86	87	90	88	93	94	94	94	93	95
		Mean	32.0	49.0	58.5	65.5	70.0	75.5	**82.0**	85.0	84.5	89.5	93.0	**95.0**	95.0	94.5	**95.5**
17	12	A	24	31	48	59	59	64	77	81	80	86	92	96	96	95	95
		B	39	65	69	70	80	85	87	90	89	93	92	94	94	92	95
		Mean	31.5	48.0	58.5	64.5	69.5	74.5	**82.0**	**85.5**	84.5	89.5	92.0	**95.0**	95.0	93.5	95.0
17	13	A	25	31	48	58	58	64	77	80	80	86	91	96	96	95	95
		B	39	65	69	69	80	85	87	89	88	93	92	94	94	93	95
		Mean	32.0	48.0	58.5	63.5	69.0	74.5	**82.0**	84.5	84.0	89.5	91.5	**95.0**	95.0	94.0	95.0
17	14	A	25	31	48	59	58	65	76	80	80	84	90	95	96	95	95
		B	40	65	69	70	80	85	87	89	88	93	92	94	93	93	95
		Mean	32.5	48.0	58.5	64.5	69.0	75.0	81.5	84.5	84.0	88.5	91.0	94.5	94.5	94.0	95.0
17	15	A	22	31	47	59	59	65	76	80	80	84	89	94	96	95	95
		B	40	65	70	69	80	85	87	89	88	93	92	94	93	92	95
		Mean	31.0	48.0	58.5	64.0	69.5	75.0	81.5	84.5	84.0	88.5	90.5	94.0	94.5	93.5	95.0
17	16	A	22	30	47	59	59	65	76	80	80	84	88	94	95	95	95
		B	40	65	70	69	80	85	87	89	88	93	92	94	93	92	95
		Mean	31.0	47.5	58.5	64.0	69.5	75.0	81.5	84.5	84.0	88.5	90.0	94.0	94.0	93.5	95.0
17	17	A	22	30	46	59	58	65	76	80	80	83	88	93	95	95	95
		B	40	65	70	69	80	85	87	89	88	93	92	94	93	92	95
		Mean	31.0	47.5	58.0	64.0	69.0	75.0	81.5	84.5	84.0	88.0	90.0	93.5	94.0	93.5	95.0

The best mean accuracy among all 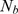 for each repetition is written in bold and the worst is underlined. An overlapped ensemble classifier becomes an ensemble classifier with naive partitioning when 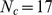 and 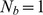. The classifier is equivalent to a single classifier when 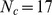 and 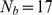.

The classification performances of ensemble classifiers with the overlapped partitioning (OSWLDA, OPCALDA and OLDA, 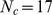, 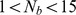) were as well as, or slightly better than those with naive partitioning (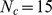 and 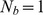) and those single classifier (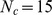 and 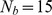) when 15300 training data were available in most sequences. The best classification performance was achieved by OSWLDA; 98% when 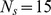, 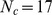, 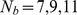. In other words, OSWLDA achieved a 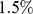 higher classification performance than the ensemble of SVMs achieved by the winner of BCI competition III data set II [23]. OSWLDA achieved about 3% improvement over single SWLDA (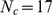, 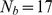). However little improvement by the ensemble classifier with the overlapped partitioning can be seen compared to the single classifier, just as the analysis of data set A using 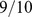 cross-validation.

## Discussion

In order to ensure the influence of the overlapped partitioning compared to traditional naive partitioning and a single classifier, classification accuracies of ensemble classifiers with those partitioning methods were compared when 900 training data were given. Two different P300-based BCI data sets were evaluated; data set A with 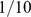 cross-validation and data set B using limited training data. The single classifier (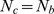 ) and the traditional ensemble classifier with naive partitioning (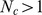 and 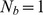) were also compared at the same time. One of three conditions for dimension reduction methods (stepwise, PCA, and none ) was also applied. The results show that OSWLDA trained on 900 ERPs achieved higher classification accuracy than the single SWLDA classifier (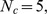 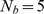) and the ensemble SWLDA classifier with naive partitioning (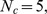 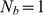) for both data sets (see Tables 4 and 6). More specifically, the proposed OSWLDA learned on 900 ERPs achieved a 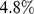 higher accuracy than the single SWLDA for data set A (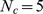, 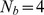, 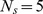) and 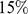 higher than the single SWLDA for data set B (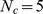, 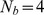, 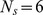), where the single SWLDA is an established and commonly used classification algorithm for P300-based BCI.

The performance improvement of proposed classifiers trained on 900 ERPs was due to the mutual effect of the overlapped partitioning and the dimension reduction. In the statistical analysis of data set A using 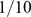 cross-validation, the main effects of the intensification sequence, degree of overlap (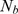), dimension reduction conditions, and their interactions were significant. According to the results shown in Figure 8 (c), indeed, the overlapped ensemble LDA classifier without dimension reduction (OLDA) did not achieve higher classification accuracies than a single LDA classifier (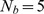) in many cases. Applying a dimension reduction method in itself is a solution to improve the classification performance of the ensemble classifier with naive partitioning. However, as shown in Figures 8 (a) and (b), when 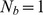, the dimension reduction method alone did not improve the classification accuracy as compared to their single classifiers. On the other hand, as also shown in Figures 8 (a) and (b), the overlapped ensemble LDA classifier together with the stepwise method (OSWLDA, 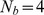) or PCA (OPCALDA, 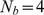) achieved higher classification accuracy than their single classifiers (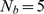). This tendency was obvious, especially for OSWLDA. Thus, the improvement in the classification accuracy was due not only to the dimension reduction or partitioning method by themselves but also to their mutual effects. Taking this into consideration, the overlapped partitioning method, together with the dimension reduction method, effectively improved the classification performance of P300-based BCI.

The performance improvement of the proposed classifiers compared to the single classifier was small when a large amount of training data were provided. However, the classification performances of proposed classifiers trained on a large amount of data were high enough to achieve 99.6% for data set A (see Table 5) and 98% for data set B (see Table 9). In those cases, however, a major performance improvement caused by overlapped partitioning was not confirmed. This was because the given training data were large enough so that the overfitting problem should not occur in most cases. Thus the advantage of overlapped partitioning can be seen when a small amount of high-dimensional training data were provided such as for the analysis of data set A using 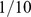 cross-validation and the data set B with limited training data.

We suggest to use the conventional cross-validation to find the optimal overlapping ratio 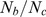 before an online experiment. However the method prolongs the training time for the classifier. Instead of that, we also suggest to use 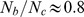 (e.g., 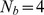 and 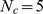) because it showed suboptimal results for both data sets. In the small training data case (900 ERP data), OSWLDA and OPCALDA with 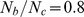 (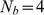 and 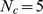) was suboptimal for both data sets A and B, but OLDA with 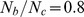 performed as well only for data set A. In the large training data case, OSWLDA, OPCALDA and OLDA with 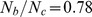 (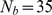 and 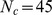) evaluated on data set A and with 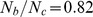 (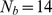 and 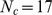) evaluated on data set B achieved reasonable classification accuracies. In this way, the overlapping ratio 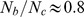 was suboptimal and it can be employed to avoid using the cross-validation.

This study first showed that the ensemble LDA classifiers with conventional naive partitioning were not effective compared to the single LDA classifier and the ensemble classifier with overlapped partitioning when 900 training data were given. This result implies that the ensemble LDA classifier with naive partitioning requires a longer training session to obtain more than 900 training data before an online experiment. It should be noted that 900 training data were the smallest used for the evaluation of the ensemble classifier to date. In contrast, the ensemble classifiers with the proposed overlapped partitioning method showed a significant improvement in the classification accuracy, which was even better than a single classifier when the stepwise method or PCA was applied for dimension reduction. Thus, overlapped partitioning was shown to be more practical than naive partitioning when the given training data were small (e.g., 900 training data).

The performance deterioration of the ensemble LDA classifiers with naive partitioning may be due to the poor estimation of the covariance matrices of LDA weak learners. Such performance deterioration can be seen in the results of OLDA on data set A using 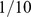 cross-validation (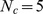, 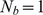), OLDA on data set A using 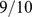 cross-validation, OSWLDA and OPCALDA on data set B with limited training data (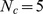, 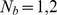), OLDA on data set B with limited training data, and OLDA on data set B with full training data (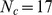, 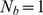). The problem can be seen when 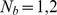 because a small amount of training data were provided to the weak learners (see Tables 2 and 3). Regarding data set B, 900 training data were not sufficient to train weak learners of OLDA (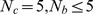 with limited training data and 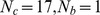 with full training data). Compared to data set A, data set B seems to require larger training data because the EEG data of data set B were higher dimensional (896 dimension). Estimated covariance matrices are imprecise when a small amount of high dimension training data are given [22]. Johnson and Krusienski first evaluated the classification performance of the ensemble SWLDA classifier with naive partitioning [27]. They evaluated the algorithm by changing the number of classifiers (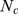 was changed while 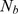 was fixed to 1). In addition, three weighting methods for the ensemble classifier were evaluated. As a result, they found that the ensemble SWLDA classifier showed better performance than the single SWLDA classifier, depending on participants, though the statistical difference was not revealed. They also discussed that the classification performance was decreased when 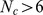 and 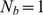 because the amount of training data for a weak learner becomes small. We consider that a similar problem arose in the application of the ensemble classifier with overlapped partitioning when 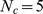 and 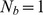, which is similar to their conditions. Such a problem can be avoided by applying the overlapped partitioning together with a dimension reduction method.

The ensemble classifiers with overlapped partitioning trained on 900 ERPs showed better classification performances than a single classifier in the middle intensification sequence condition in the offline analysis. According to Figure 8 (a), OSWLDA (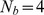) achieved higher classification accuracy than the single SWLDA classifier (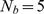 ) among 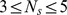. In contrast, the OPCALDA (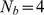) showed higher classification accuracy than the single PCA LDA classifier (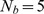 ) when 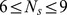. This result implies that the ensemble classifier with overlapped partitioning was beneficial in the middle number of the intensification sequence. 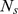 decides the terms to compute the score for decision making according to Equation 2. The performance saturation can be seen as the 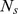 become larger while the classification performance was not precise when 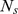 was smaller. In both cases, differences of those classification performances were hard to confirm. This might explain why the classification performance difference was obvious in the middle number of sequences.

The selection of the number of the intensification sequence in an online P300-based BCI experiment depends on the applications of the BCI system. One criterion is the information transfer rate (ITR), which takes the accuracy, number of outputs, and output time (the number of sequences) into consideration [35]. OSWLDA on data set A using 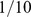 cross-validation (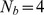) achieved the highest ITR (15.7 bits per minute) at 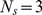, although only a 71.4% accuracy was expected in an online experiment. On the other hand, accuracy must be prioritized, for example, when the BCI is used to provide precise control of a robotic manipulator that could be dangerous. To decide parameters such as the number of intensification sequences, we should consider what kind of criterion (accuracy, speed, or ITR) should be optimized in terms of BCI applications.

Determining the amount of training data also decides an expected online classification accuracy. If the system needs over 70% mean classification accuracy, only 900 training data are required. In case that over 95% mean accuracy is required, a large amount of training data should be prepared. Most BCI applications do not usually require over 95% classification accuracy because they are free from danger. Thus 900 training data are sufficient to achieve over 70% mean accuracy for most applications of BCI.

We would like to emphasize that the ensemble classifiers with overlapped partitioning required less training data than that with naive partitioning. OSWLDA and OPCALDA performs better than the ensemble classifier with naive partitioning enough to achieve over 90% classification accuracy using only 900 training data. Especially the mean classification accuracy of OSWLDA (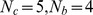) with the small training data achieved as well as that of ensemble SWLDA with naive partitioning (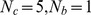) for data set A. In this way the ensemble classifier with overlapped partitioning require less training samples than that with naive partitioning so that it might be useful to do away with expensive experiments.

In this research, the PCA and stepwise method were applied as a dimension reduction. The PCA and the stepwise method have different statistical properties; PCA finds the projection that maximizes the data variance while the stepwise method selects spatiotemporal variables. Although no great difference was found in the classification accuracy for data set A using 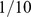 and 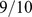 cross-validation and data set B with full training data, OSWLDA showed better performance than OPCALDA for data set B with limited training data. In this way, the stepwise method was robust for both P300-based BCI data sets. The difference between the two also appears in the online/offline test computational cost; the stepwise method requires a smaller processing burden than PCA because the stepwise method in the test case does not use data projection. The difference will be more obvious when 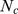 becomes large. Considering the computational cost, the stepwise method is preferable in case a large number of classifiers are required.

In future research, LDA with shrinkage [22] or Bayesian LDA [32] will be applied to the ensemble classifier with overlapped partitioning. These two methods estimate covariance matrices in different ways so that LDA in itself becomes robust against a lack of training data. Thus, it may be possible to achieve better classification accuracy with a smaller amount of training data by applying the two methods. The proposed ensemble classifiers with overlapped partitioning may be applicable to other types of BCIs such as an event-related desynchronization/synchronization (ERD/ERS)-based BCI [42]. In fact, some ensemble classifiers for ERD/ERS-based BCIs were evaluated [43] and our proposed overlapped ensemble classifiers might also be applicable. Moreover, the ensemble classifier with the overlapped partitioning can be used in other pattern recognition problems, e.g., a cancer classification [44] or fMRI data analysis [45]. Furthermore, clustering algorithms such as k-means clustering [46] could be used for a new overlapped partitioning of the ensemble classifiers. By clustering the data with overlaps, classifiers that perform well for specific features can be trained. Thus, the clustered partitioning with overlaps may show an even better classification performance.

## Conclusion

In this study, ensemble LDA classifiers with the newly proposed overlapped partitioning method were evaluated on our original P300-based BCI data set and the BCI competition III data set II. In the comparison, the classifiers were trained on limited training data (900) and large training data. The ensemble LDA classifier with traditional naive partitioning and the single classifier were also evaluated. One of three conditions for dimension reduction (stepwise, PCA, or none ) was applied. As a result, the ensemble LDA classifier with overlapped partitioning and the stepwise method (OSWLDA) showed higher accuracy than the commonly used single SWLDA classifier and the ensemble SWLDA classifier when 900 training data were available. In addition, the ensemble LDA classifiers with naive partitioning showed the worst performance for most conditions. We suggest to use the stepwise method as a dimension reduction for the online implementation. In future research, the LDA with shrinkage or Bayesian LDA will be applied to the ensemble classifier with overlapped partitioning.
